# Coronary Aneurysm Occurring Late after Drug-Eluting Stent Implantation

**DOI:** 10.5402/2011/367512

**Published:** 2011-05-11

**Authors:** Etan Abergel, Ariel Roguin

**Affiliations:** Department of Cardiology, Rambam Medical Center, B. Rappaport-Faculty of Medicine, Technion-Israel Institute of Technology, Haifa 31096, Israel

## Abstract

Drug-eluting stents may affect the normal healing process of the vessel wall and the remodeling process and may lead to late stent malapposition (LSM). The known incidence of this phenomen originates from short-term angiographic follow-up studies. We describe a case report of very late stent malapposition and marked positive vessel remodeling 3 years after sirolimus-eluting coronary stent implantation. Angiography performed one year after stent implantation was normal. Thus, the abnormalities developed sometime between years 1 and 3. The cause is unknown, but it is reasonable to suggest a local effect of the medication/polymer of the stent. LSM rate and aneurysmal formation is higher in DES than in BMS and may be associated with increased risk for late stent thrombosis. Currently, the risk of very late stent thrombosis after DES implantation is of major concern. As observed in this case report, LSM might occur and develop very late. This has significant consequences especially to the many asymptomatic patients with DES implanted many years ago and the recommendation of dual antiplatelet therapy. More studies with late and very late follow up are needed to better define this finding, its mechanism, how to avoid it, and how to treat it properly.

## 1. Introduction

Positive remodeling, regression of neointimal proliferation, and late malapposition were described with brachytherapy [1]. Major attention recently has been focused on late complications after drug-eluting stents (DESs) implantation [2–6]. DES may affect the normal healing process of the vessel wall and the remodeling process and may lead to late stent malapposition (LSM) [7–9]. We describe a case report of very late stent malapposition and marked positive vessel remodeling 3 years after sirolimus-eluting coronary stent (SES) implantation.

## 2. Case Report

A-45year-old patient with history of hypercholesterolemia and no diabetes mellitus was admitted to our hospital on April 2005 for elective coronary angiography for typical angina pectoris with Canadian Cardiovascular Society (CCS) score of 3. Three-vessel disease was diagnosed: 75% stenosis of the mid-left anterior descending artery (LAD), 75% stenosis of the mid-left circumflex artery (LtCx), and a chronic total occlusion (CTO) of the mid right coronary artery (RCA). Staging procedure was decided and a 3 × 18 mm SES was implanted to the mid-LtCx in the index procedure. A-week later another 3 × 18 mm SES was implanted to the mid LAD. The CTO was successfully treated and 2 bare metal stents (BMSs) were implanted in the mid-RCA (Motion 3 × 30 mm and Clearflex 2.5 × 13 mm). On April 2006, a year later, the patient was readmitted for typical anginal syndrome. Angiography revealed that both SESs in the LAD and in the LtCx were patent. The SESs were patent with very minimal angiographic positive remodeling around each of the SES stents. There was focal in-stent restenosis in the BMS in the RCA. Due to concerns about therapy compliance, we decided to treat the patient with balloon dilatation only without stent implantation.

**Figure 1:**
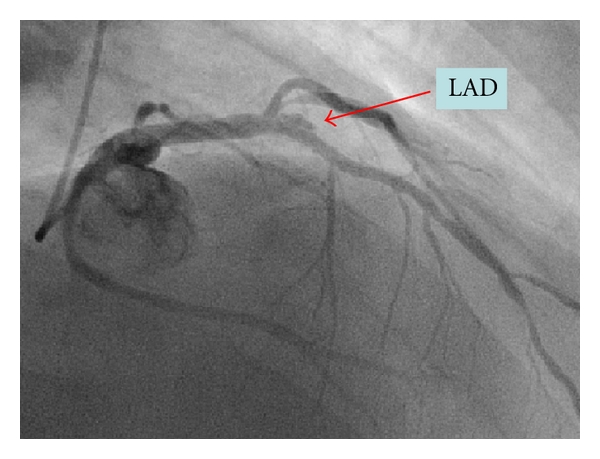
Mid-LAD aneurysm.

**Figure 2:**
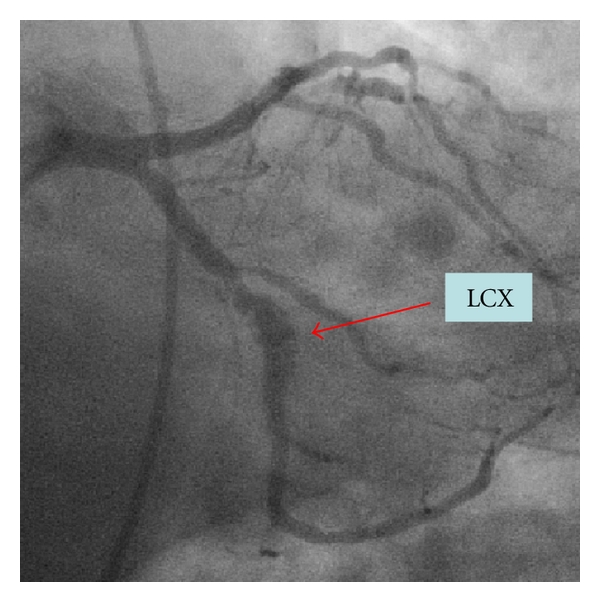
Mid-LtCx aneurysm.

**Figure 3:**
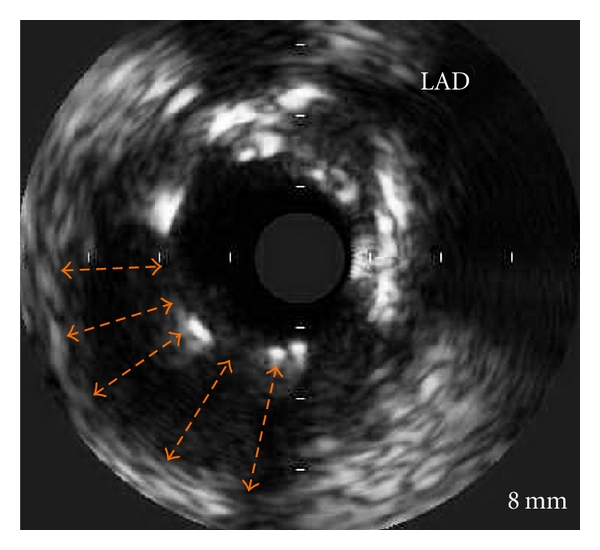
IVUS of LAD showing the stent struts and the “black hole” which was formed (dashed orange lines).

**Figure 4:**
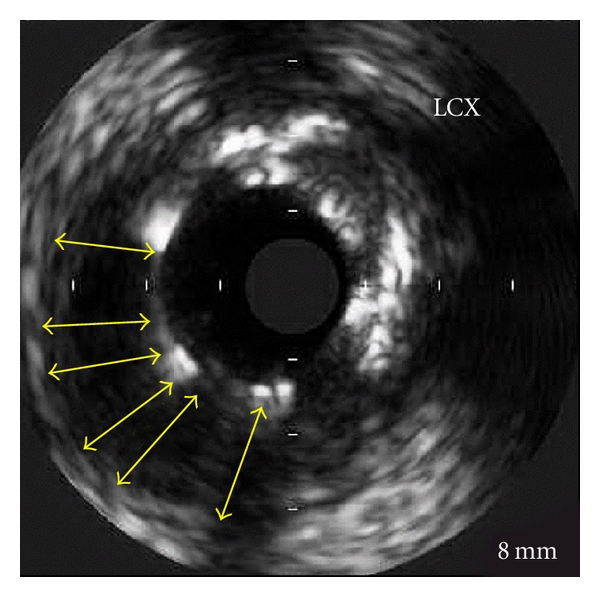
IVUS of LtCx showing the stent struts and the “black hole” which was formed (yellow lines).

In 2008, three years after the index hospitalization, due to angina recurrence, the patient underwent another coronary angiography. The RCA stents were patent. However, as compared to the images obtained two years prior, a significant positive remodeling evolved and formed a large peristent aneurysmal defect around both SES in the mid-LAD and in the mid-LtCx (Figures 1 and 2). Intravascular ultrasound (IVUS) to the LAD and the LtCx confirmed the positive remodeling and the presence of very LSM in both SESs (Figures 3 and 4).

## 3. Discussion

We report a case report of very late LSM with SES. There was deterioration in the angiographic appearance from 1 year to 3 years after stenting. Thus, the abnormalities developed sometime between years 1 and 3. The cause is unknown, but it is reasonable to suggest a local effect of the medication/polymer of the stent. 

LSM rate and aneurysmal formation is higher in DES than in BMS [3–5]. The mechanism of this phenomenon remains unclear [2–9]. Several hypotheses have been suggested; the first is that the antiproliferative drug may exclude the proliferation of tissue in the void initially present between the struts and the vessel wall. Second, the drug may induce apoptosis or necrosis, creating a new space between the vessel wall and the struts initially well apposed. The third suggests that the drug may prevent myoblasts proliferation in an organized thrombus which will eventually dissolve creating an empty space between the struts and the vessel wall.

Previous reports demonstrated aneurysmal formation at the edges of the stent in intravascular brachytherapy [1], as well as after BMS implantation. Serial IVUS analysis from the SIRIUS trial found an 8.7% incidence of LSM after SES implantation, with LSM being observed primarily in the middle portion of the stent. The same location was observed in the present case report. The different locations of aneurysmal formation indicate involvement of different biologic processes in the focal positive remodeling and subsequent development of LSM in SES. LSM as in our case may occur due to profound localized inhibition of neointimal formation in disease-free segments of the vessel, delaying early reparative events that usually enable the vessel wall to incorporate a stent. Flow dynamics in these segments may also favor positive remodeling of the artery at sites of delayed neointimal formation, resulting in late-acquired aneurysmal formation.

Other hypothesis includes extensive acute vessel damage during the initial procedure, hypersensitivity reactions, infectious processes, and phenomena resembling extreme cases of late-acquired malapposition [5–9]. The large numb er of hypothesis suggests that we do not know the pathogenesis, and worse than that we do not know how to prevent or treat this finding.

What is the prognosis of patients with this finding? According to the RAVEL and SIRIUS study followup at 4 years [4] in the SES population only, there was a non statistically significant difference in MACE between the patients with or without LSM (11.1% versus 16.3%, *P* = .48), and the authors concluded that LSM appears to be an IVUS finding without significant impact on the incidence of major adverse cardiac events even during long-term followup. 

In contrast, Hassan et al. [5] in a recent meta-analysis compared BMS to DES LSM rates and long-term followup in seventeen studies. The risk of LSM in patients with DES was four times higher compared with BMS (*OR* = 4.36, CI 95% 1.74–10.94). The risk of late stent thrombosis in patients with LSM was higher compared with those without LSM (*OR* = 6.51, CI 95% 1.34–34.91). The authors concluded that LSM is strongly increased after DES implantation compared with BMS, and furthermore, LSM seems to be associated with late and very late stent thrombosis. 

Currently, the risk of very late stent thrombosis after DES implantation is of major concern. It seems that patients with LSM are at increased risk for this adverse effect. As observed in this case report, LSM might unfortunately occur and develop very late. This has significant consequences especially to the many asymptomatic patients with DES implanted in their coronaries several years ago. We do not know whether such LSM develops late many years after DES implantation. We should maybe consider a longer dual antiplatelet therapy. More studies with late and very late followup are needed to better define this finding, its mechanism, how to avoid it and how to treat it, properly.
